# Dual Phospho-CyTOF Workflows for Comparative JAK/STAT Signaling Analysis in Human Cryopreserved PBMCs and Whole Blood

**DOI:** 10.21769/BioProtoc.5512

**Published:** 2025-11-20

**Authors:** Ilyssa E. Ramos, Brynja Matthiasardottir, Teresa S. Hawley, Kyu Lee Han, Michal Toborek, Iyadh Douagi, Georgette N. Jones, James M. Cherry

**Affiliations:** 1 Protein and Chemistry Section (PCS), Research Technologies Branch (RTB), National Institute of Allergy and Infectious Diseases, Bethesda, MD, USA; 2 Center for Human Immunology, Autoimmunity, and Inflammation (CHI), National Institute of Allergy and Infectious Diseases, Bethesda, MD, USA; 3 Department of Biochemistry and Molecular Biology, University of Miami Miller School of Medicine, Coral Gables, FL, USA; 4 National Human Genome Research Institute (NHGRI), Bethesda, MD, USA; 5 Computational Biology, Bioinformatics, and Genomics, Biological Sciences, University of Maryland, Baltimore, MD, USA; 6 Flow Cytometry Section, Research Technologies Branch (RTB), National Institute of Allergy and Infectious Diseases, Bethesda, MD, USA

**Keywords:** Mass cytometry, Helios, Phospho-CyTOF, Whole blood, Cryopreserved PBMCs (cPBMCs), JAK/STAT signaling, Type I/II interferon, IL-21, Cytokine stimulation, Pleiotropic immune cell signaling

## Abstract

Protein phosphorylation is a dynamic post-translational modification that regulates fundamental processes, including signal transduction, cell proliferation, differentiation, and effector function of immune cells. The Janus Kinase/Signal Transducer and Activator of Transcription (JAK/STAT) pathway is a key mediator of cytokine responses, essential for maintaining immune cell homeostasis and determining cell fate across diverse immune subsets. Dysregulation of JAK/STAT signaling has been linked to a broad spectrum of pathologies, including monogenic immune disorders, autoimmunity, and cancer. Platforms facilitating single-cell analysis of protein phosphorylation offer the ability to reveal subtle signaling defects and dissect the pleiotropy in cellular composition and phosphorylation status, providing insights into immune phenotype and function, while identifying potential therapeutic targets. While an application of cytometry-by-time-of-flight, termed phospho-CyTOF, has proven invaluable for studying protein phosphorylation in cryopreserved peripheral blood mononuclear cells (cPBMCs), its application is limited by cell loss and signaling artifacts stemming from isolation and cryopreservation. Conversely, whole blood (WB) approaches, preserving the native immune cell composition and signaling context, offer a more physiological representation but necessitate robust and consistent protocols for broad application. Herein, we present optimized dual phospho-CyTOF workflows tailored for both cPBMCs and whole blood, building upon established protocols for cytokine stimulation of both samples. These workflows facilitate comprehensive, high-dimensional profiling of JAK/STAT signaling in response to pleiotropic cytokines such as Type I interferons (IFN-α), Type II interferons (IFN-γ), and Interleukin-21 (IL-21). By leveraging CyTOF's capacity for high-dimensional profiling using pure heavy metal–labeled antibodies, these protocols aim to identify pathway-specific alterations in STAT phosphorylation across major immune subsets that may be overlooked by traditional flow cytometry. Together, these optimized dual workflows provide scalable, translationally relevant tools for dissecting the subtle and differential JAK/STAT-driven immune responses in both clinical and research settings, while also being compatible with the simultaneous assessment of crosstalk with alternative immune cell signaling pathways.

Key features

• This method enables multiplexed detection of 20 surface markers and STAT phosphorylation to resolve subsets and interrogate diverse JAK/STAT signaling.

• Whole blood workflow supports rapid “vein-to-tube” processing and fixation, preserving native signaling, immune cell composition, and fragile myeloid subsets.

• Designed for users with CyTOF expertise who are proficient in cytometry workflows involving surface and intracellular staining, fixation, and phospho-epitope preservation across immune subsets.

• Applicable to clinical immunomonitoring, pharmacodynamic studies (i.e., JAK inhibitors), and biomarker discovery in immune dysregulation.

## Graphical overview


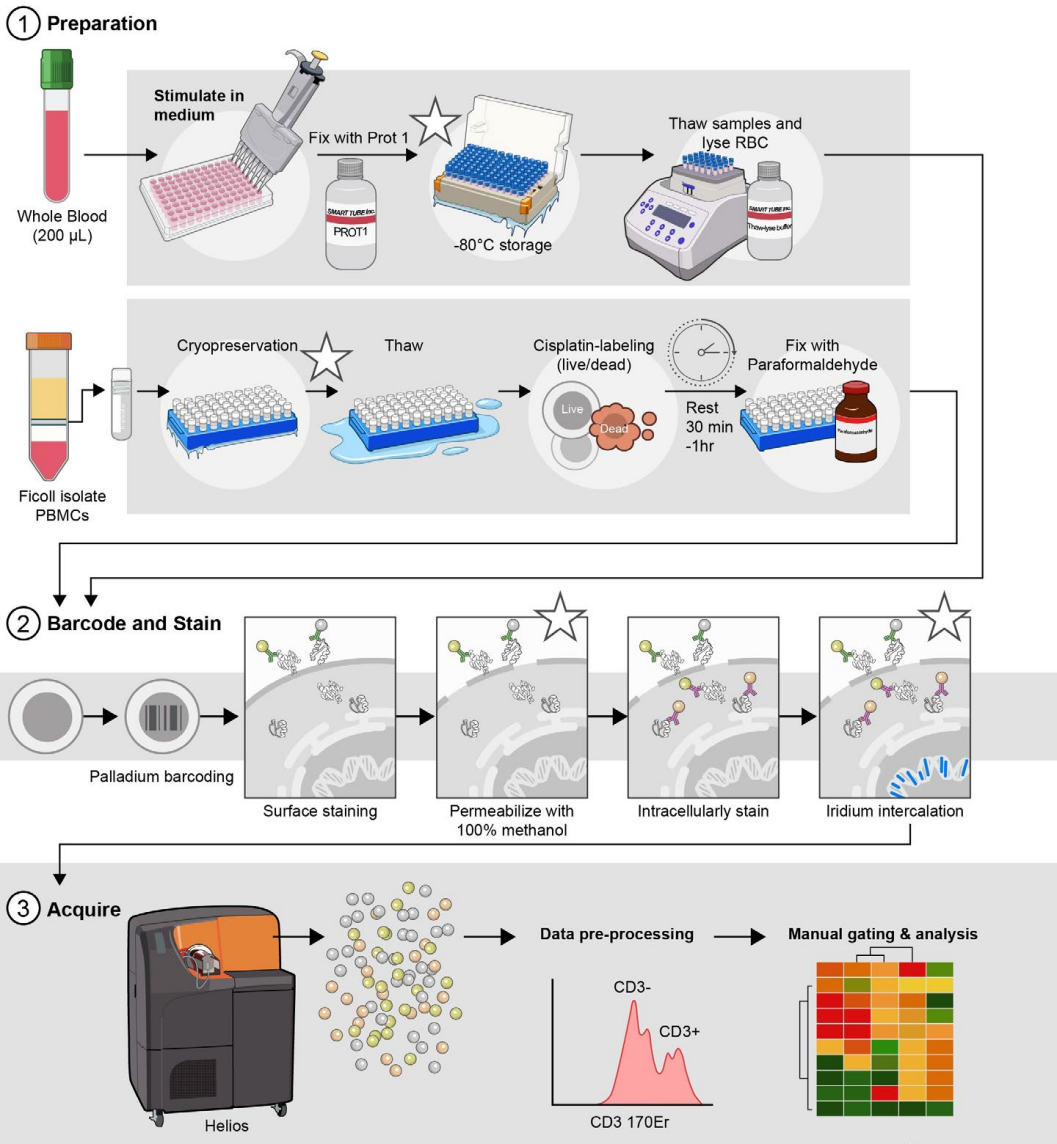


**Optimized parallel workflows for assessing immune cell signaling via Phospho-CyTOF.** (1) Sample preparation and stimulation: cryopreserved peripheral blood mononuclear cells (cPBMCs) or whole blood are stimulated with IFN-α, IFN-γ, or IL-21 to induce specific JAK/STAT phosphorylation events. Immediately following stimulation, cells are fixed to preserve phosphorylation states: cPBMCs with 2.2% paraformaldehyde (PFA) and WB samples with Prot1 fixative (Smart Tube, Inc.). Whole blood samples then undergo erythrocyte lysis using thaw/lyse solution (Smart Tube, Inc.). (2) Barcoding, staining, permeabilization, intracellular staining, and iridium intercalation: Both cPBMC and lysed WB samples are subsequently palladium-barcoded for multiplexing, followed by surface staining to identify major immune cell subsets. To facilitate intracellular staining, cells are permeabilized overnight in 100% methanol. The following day, samples are rehydrated and stained for intracellular phospho-proteins (e.g., pSTAT1, pSTAT3, pSTAT4, pSTAT5, and pSTAT6) and other intracellular markers (e.g., pP38, pERK1/2, IKβα). To exclude doublets during acquisition, cells are incubated in iridium intercalation solution. (3) Sample acquisition: Prior to acquisition, samples are resuspended at an optimized concentration to achieve approximately 350 events per second (± 50 events per second) and diluted 1:10 with EQ Four Element Calibration Beads (Standard BioTools) in cell acquisition solution plus (CAS^+^). These data are acquired with a Helios mass cytometer. *Indicates potential stopping points in the protocol.

## Background

Protein phosphorylation is a reversible post-translational modification that plays a central role in regulating immune cell signaling, activation, differentiation, and effector functions [1]. This process is orchestrated by kinases and phosphatases, which add or remove phosphate groups from serine, threonine, or tyrosine residues on intracellular signaling proteins [2]. Dysregulation of these signaling events is linked to the pathogenesis of various diseases, including autoimmune disorders, autoinflammatory syndromes, immunodeficiencies, allergies, and malignancies.

Intracellular signal transduction is a crucial cell communication process that transfers extracellular signals regulating activation, survival, proliferation, and differentiation in immune cells [3]. Pathway activation is regulated by the binding of cytokines, inflammatory mediators, hormones, and extracellular messengers to cell surface receptors, which initialize signal transducers (i.e., kinases and transcription factors) [4] to drive cell phenotype and function. The strict temporal and spatial regulation of activated signal transducers drive major cellular decisions, such as cell cycle checkpoints, apoptosis, and cytoskeletal reorganization [5]. Studying changes in cellular communication methods (i.e., phosphorylation) under various conditions can provide a deeper understanding of underlying biological processes, emphasizing the importance of understanding how these signaling pathways are stimulated and regulated [6].

**Figure 1.**
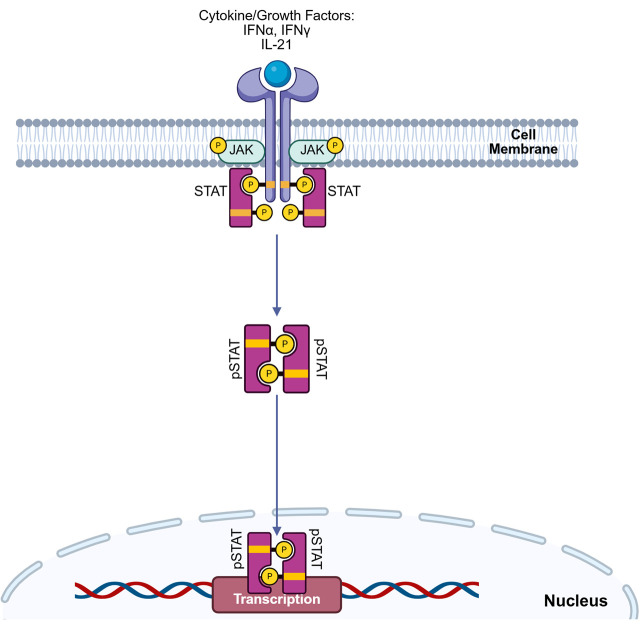
Extracellular receptor engagement triggers intracellular STAT phosphorylation in immune cells. The binding of extracellular cytokines, growth factors, or interferons to their cognate receptors initiates a rapid intracellular signaling program. This phenomenon triggers autophosphorylation of Janus-associated kinases (JAK), followed by sequential docking and phosphorylation/activation of signal transducer and activator of transcription (STAT) proteins. Phosphorylated STATs dimerize and translocate into the nucleus, driving transcriptional programs that lead to pro- or anti-inflammatory phenotypes in immune cells.

The Janus Kinase/Signal Transducer and Activator of Transcription (JAK/STAT) pathway is a crucial signaling cascade that orchestrates a diverse array of cellular processes essential for immune homeostasis and host defense. Due to its pleiotropy, this pathway mediates the effects of multiple cytokines (>50), interferons, and growth factors that dictate cell fate, proliferation, migration, metabolism, differentiation, and effector function across all immune subsets [7] (Figure 1). In mammals, the JAK family comprises four proteins, JAK1-3 and TYK2, whereas the STAT family contains seven proteins (STAT1-4, STAT5A/5B, and STAT6). Even within the same family, different cytokines engage unique JAK-STAT combinations, resulting in distinct transcriptional programs that modulate cellular responses [8]. For example, Type I interferons (IFN-α), Type II interferons (IFN-γ), and Interleukin-21 (IL-21) are potent immune modulators that typically activate diverse, sometimes overlapping, STAT molecules to drive biological outcomes, from antiviral and anti-tumor immunity to T follicular helper cell differentiation and B-cell responses. Mounting evidence elucidates the role of dysregulated JAK/STAT signaling in inborn errors of immunity (IEI) and cancer, underscoring the need to effectively interrogate this pathway in complex biological samples.

A thorough assessment of JAK/STAT signaling is critical for understanding immune dysregulation in disease and in guiding the development and deployment of targeted therapies. Traditional methods for studying protein phosphorylation, such as western blot and ELISA, have limitations due to their bulk nature, low throughput, and inability to resolve signaling at the single-cell level [8]. Fluorescence-based phospho-flow cytometry enables multiplexed, single-cell analysis; however, it faces constraints such as spectral overlap, compensation issues, and autofluorescence [9]. Mass cytometry, also known as cytometry by time-of-flight (CyTOF), has emerged as a state-of-the-art technology that utilizes heavy metal–conjugated antibodies for the detection of proteins, thereby circumventing the limitations of flow cytometry and enabling simultaneous measurements of over 40 parameters per cell, including both surface markers and intracellular phospho-epitopes [10,11]. This high-dimensional method, phospho-CyTOF, is a powerful tool for the simultaneous assessment of STAT phosphorylation across key immune cell subsets with single-cell resolution.

In translational immunology, a significant challenge is the choice of the biological sample matrix, i.e., cryopreserved peripheral blood mononuclear cells (cPBMCs) vs. whole blood (WB). Despite the invaluable advantage of longitudinal sample banking and batch processing, the isolation of PBMCs and cryopreservation can induce cellular stress, alter basal phosphorylation states, and potentially impact cellular responsiveness [12]. Conversely, whole blood stimulation offers a more physiologically relevant "vein-to-tube" approach, maintaining a full cellular repertoire that preserves cell-to-cell interactions that occur in vivo, despite presenting logistical hurdles for large-scale, multi-center studies.

To address this fundamental question, we present optimized dual phospho-CyTOF workflows that can be used to investigate the pleiotropic effect of cytokine stimulation on JAK/STAT signaling in immune cells from both cryopreserved PBMCs [13,14] and whole blood [15]. These protocols enable concurrent immunophenotyping and phospho-protein profiling that is compatible with high-dimensional data analysis pipelines. To ensure translational relevance, we adapted a protocol introduced in both Fernandez and Maecker’s 2015 *Bio-Protocols* for cPBMCs [13,14] and whole blood [15]. Specifically, we improve upon cell loss that is experienced following repeated fixation and the methanol permeabilization steps, implement palladium barcoding to limit batch variability in staining, and expand the intracellular panel to include six of seven STAT molecules for more comprehensive functional profiling. The overarching goal is to directly compare and refine a protocol for each sample type, thereby aiding researchers in leveraging the strengths and weaknesses of both approaches to address their specific biological question. Furthermore, by mapping differential phosphorylation of STAT1, STAT3, STAT4, STAT5, and STAT6 in response to the cytokines IFN-α, IFN-γ, and IL-21, across major immune subsets, this protocol provides invaluable insights into the nuanced and context-dependent nature of JAK/STAT signaling in human immunity for immediate clinical application. Understanding differential phospho-STAT responses across sample types is crucial for designing robust immunomonitoring studies and for precisely interpreting results in diverse research and clinical settings that may contribute to understanding human immunity and driving therapeutic strategies in instances of dysregulation.

## Materials and reagents

**Biological materials**

1. Human whole blood preserved in sodium heparin tubes (NIH Clinical Center Blood Bank)

2. Cryopreserved peripheral blood mononuclear cells (cPBMCs) (NIH Clinical Center Blood Bank)

**Reagents**

1. IFNα (PBL, catalog number: 11105-1)

2. IFNγ (BD Biosciences, catalog number: 554617)

3. Lipopolysaccharide (LPS) from *Salmonella enterica* serotype enteritidis γ-irradiated, BioXtra, suitable for cell culture (Sigma-Aldrich, catalog number: L7770-1MG)

4. IL-21 (Fisher Scientific, catalog number: PHC0214)

5. RPMI 1640 medium, GlutaMAX^TM^ supplement (Thermo Fisher Scientific, catalog number: 61870036)

6. Heat-inactivated fetal bovine serum (FBS) (Thermo Fisher Scientific, catalog number: A5670801)

7. Dulbecco’s modified PBS without Ca^2+^/Mg^2+^ (Thermo Fisher Scientific, catalog number: 14190094)

8. Penicillin-streptomycin (10,000 U/mL) (pen/strep) (Thermo Fisher Scientific, catalog number: 15140122)

9. 16% formaldehyde solution (w/v) methanol-free (Thermo Fisher Scientific, catalog number: 28906)

10. Cell-ID Cisplatin-^195^Pt (Standard BioTools, catalog number: 201195)

11. Pierce Universal nuclease for cell lysis, 25 kU (nuclease inhibitor) (Thermo Fisher Scientific, catalog number: 88701)

12. Prot1 (SmartTube Inc., catalog number: 501351691)

13. Thaw/lyse concentrate (SmartTube Inc., catalog number: 501351696)

14. Methanol (MeOH) ACS reagent, ≥99.8%, 1 L (Sigma-Aldrich, catalog number: 179337-1L)

15. Water, molecular biology (Quality Biological, catalog number: 351-029-101)

16. Maxpar 10× barcode perm concentrate (50 mL) (Standard BioTools, catalog number: 201057)

17. Maxpar PBS (500 mL) (Standard BioTools, catalog number: 201058)

18. Maxpar^®^ cell staining buffer (500 mL) (Standard BioTools, catalog number: 201068)

19. Cell-ID^TM^ 20-Plex Pd Barcoding kit (Standard BioTools, catalog number: 201060)

20. Cell-ID^TM^ Intercalator-Ir, 12.5 μM (500 μL) (Standard BioTools, catalog number: 201192C)

21. EQ Four Element calibration beads (100 mL) (Standard BioTools, catalog number: 201078)

22. Maxpar^®^ fix and perm buffer (100 mL) (Standard BioTools, catalog number: 201067)

23. Maxpar^®^ cell acquisition solution plus for CyTOF^®^ XT (1,000 mL) (CAS^+^) (Standard BioTools, catalog number: 201244)

24. Maxpar^®^ water (500 mL) (Standard BioTools, catalog number: 201069)

25. CyTOF tuning solution (250 mL) (Standard BioTools, catalog number: 201072)

**Solutions**

1. Thawing media (see Recipes)

2. Cisplatin labeling media (see Recipes)

3. 2× Cell-ID Cisplatin-^195^Pt working solution (see Recipes)

4. Stimulation media (see Recipes)

5. 1× thaw/lyse buffer (see Recipes)

6. 1× barcoding buffer (BC buffer) (see Recipes)

7. Cell-ID^TM^ Intercalator-Ir Solution (iridium intercalation solution) (see Recipes)

8. 1:10 EQ Four Element calibration beads (1:10 EQ4 beads) (see Recipes)

**Recipes**

**1. Thawing media**

**Table:** 

Reagent	Final concentration	Volume
RPMI 1640 medium, GlutaMAX^TM^ supplement	89%	44.495 mL
Heat-inactivated FBS	10%	5.000 mL
Penicillin-streptomycin (10,000 U/mL)	1%	0.500 mL
Pierce Universal nuclease for cell lysis	10,000×	0.005 mL
Total		50.000 mL

a. Alternatively, aliquots of stimulation media (Recipe 4) can be used as a base to directly add Pierce Universal nuclease for cell lysis.

b. Prepare thawing media immediately before use and discard remaining media once thawing is complete.

c. Warm the media in a 37 °C water bath to thaw the PBMCs.

**2. Cisplatin labeling media**

**Table:** 

Reagent	Final concentration	Volume
RPMI 1640 medium, GlutaMAX^TM^ supplement	99%	495 mL
Penicillin-streptomycin (10,000 U/mL)	1%	5 mL
Total		500 mL

a. Filter-sterilize the cisplatin labeling media through a 500 mL filtration unit with a 0.22 μm pore size.

b. Media can be stored at 4 °C for up to one month.

c. Warm the cisplatin labeling media in a 37 °C water bath to label PBMCs.

**3. 2× Cell-ID Cisplatin ^195^Pt working solution**

**Table:** 

Reagent	Final concentration	Volume
RPMI 1640 medium, GlutaMAX^TM^ supplement		9.995 mL
Cell-ID Cisplatin-^195^Pt	2,000×	0.005 mL
Total		10.0 mL

a. Prepare the 2× Cell-ID Cisplatin-^195^Pt working solution immediately before use and discard the remaining solution once labeling is complete.

b. Add 0.5 μL/mL of 2× Cell-ID Cisplatin-^195^Pt working solution to the appropriate volume of cisplatin labeling media to create a 2× Cell-ID Cisplatin-^195^Pt working solution.

**4. Stimulation media**

**Table:** 

Reagent	Final concentration	Volume
RPMI 1640 medium, GlutaMAX^TM^ supplement	89%	445 mL
Heat-inactivated fetal bovine serum (FBS)	10%	50 mL
Penicillin-streptomycin (10,000 U/mL)	1%	5 mL
Total		500 mL

Filter-sterilize the stimulation media through a 500 mL 0.22 μm filtration unit. Stimulation media can be prepared up to one month before use and stored at 4 °C.

**5. 1× Thaw/lyse buffer**

**Table:** 

Reagent	Final concentration	Volume
Water, molecular biology	NA	49.95 mL
Thaw/lyse concentrate (1,000×)	1×	0.05 mL
Total		50 mL

a. Filter-sterilize the 1× thaw/lyse buffer through a 500 mL 0.22 μm filtration unit.

b. 1× thaw/lyse buffer can be prepared and stored at room temperature (RT) for up to one month from the preparation date.

**6. 1× Barcoding buffer (BC buffer)**

**Table:** 

Reagent	Final concentration	Volume
MaxPar PBS	NA	45 mL
Maxpar 10× barcode perm concentrate	1×	5 mL
Total		50 mL

1× BC buffer can be stored at 4 °C and used up to one month from the preparation date.

**7. Iridium intercalation solution**

**Table:** 

Reagent	Final concentration	Volume
Maxpar^®^ fix and perm buffer	NA	3.998 mL
Cell-ID^TM^ Intercalator-Ir, 12.5 μm	2,000×	0.002 mL
Total		4.000 mL

a. The iridium intercalation solution should be prepared immediately before use and stored on ice.

b. Iridium intercalator stock should be aliquoted immediately after thawing.

c. After a second thaw, aliquots of iridium intercalator stock should be discarded.

**8. 1:10 EQ4 beads**

**Table:** 

Reagent	Final concentration	Volume
Maxpar^®^ cell acquisition solution plus for CyTOF^®^ XT (CAS^+^)	NA	9 mL
EQ Four Element calibration beads	10×	1 mL
Total		10 mL

a. The EQ Four Element calibration beads require vigorous shaking by hand for at least 30 s prior to use.

b. Do not vortex the EQ Four Element calibration beads, as it fails to resuspend the beads into solution.

c. The 1:10 EQ Four Element calibration beads in CAS^+^ (1:10 EQ4 Beads) should be prepared immediately before use and stored on ice.

**Laboratory supplies**

1. 25 mL sterile reservoirs (Thermo Fisher Scientific, catalog number: 95128095)

2. DNA LoBind tube 2.0 mL (Eppendorf, catalog number: 022431048)

3. Safe-Lock tubes 1.5 mL, natural (Eppendorf, catalog number: 022363212)

4. Dry Ice

5. Matrix tube 0.5 mL 2D screw tubes PP, V-bottom, w/clear caps (Thermo Fisher Scientific, catalog number: 3744)

6. Matrix tube 0.5 mL 2D screw tubes PP, V-bottom, w/blue caps (Thermo Fisher Scientific, catalog number: 3744BLU)

7. Matrix tube 0.5 mL 2D screw tubes PP, V-bottom, w/pink caps (Thermo Fisher Scientific, catalog number: 3744PIN)

8. Matrix tube 0.5 mL 2D screw tubes PP, V-bottom, w/green caps (Thermo Fisher Scientific, catalog number: 3744GRE)

9. Matrix tube 0.5 mL 2D screw tubes PP, V-bottom, w/yellow caps (Thermo Fisher Scientific, catalog number: 3744YEL)

10. Millipore^®^ Stericup^®^ quick release vacuum filtration system (Millipore Sigma, catalog number: S2GPU10RE)

11. 14 mL polystyrene round-bottom tube (Corning, catalog number: 352051)

12. 5 mL polystyrene round-bottom tube with cell strainer cap (Falcon, catalog number: 352235)

13. 50 mL polypropylene conical tube 30 × 115 mm style (Corning, catalog number: 352070)

14. RTS LTS 1,200 μL filter 768/4 tips (Rainin, catalog number: 17007084)

15. RTS LTS 1,000 μL filter 768/4 tips (Rainin, catalog number: 17014967)

16. RTS LTS 200 μL F 960A/10 (Rainin, catalog number: 30389239)

17. Dualfilter 200 μL, PCR clean/sterile (Eppendorf, catalog number: 022491296)

18. P20 LTS pipette tips (Rainin, catalog number: 17014392)

19. Sterile 5 mL serological pipette (VWR Scientific, catalog number: 612-3702)

20. Sterile 10 mL serological pipette (VWR Scientific, catalog number: 89130-898)

21. Plate seal (VWR Scientific, catalog number: 734-4000)

22. 96-well deep well 2 mL plate (Fisher brand, catalog number: 12-566-121)

23. 96-well V-bottom plate with lid, polystyrene, TC-treated (Corning, catalog number: 3894)

24. 96-well U-bottom plate with lid, polystyrene, TC-treated (Corning, catalog number: 3799)

25. C-Chip (inCyto, catalog number: DHC-N01-5)

## Equipment

1. Helios CyTOF (Standard BioTools, model: PN 400250 A7)

2. Cellaca MX high-throughput cell counter (Revvity, catalog number: MX-AOPI)

3. Thermo Sorvall Legend XTR refrigerated centrifuge (Marshall Scientific, catalog number: TSO-LEGXTR)

4. Corning^TM^ mini microcentrifuge, 100–240V (Fisher Scientific, catalog number: 07-203-954)

5. -86 °C upright freezer (Phcbi, catalog number: MDFDU702VHPA)

6. -20 °C K2 scientific laboratory undercounter freezer (Fisher Scientific, catalog number: K204SDF)

7. 4 °C TSX Series high-performance lab refrigerator (Thermo Fisher Scientific, catalog number: TSX3005SD)

8. Eppendorf^TM^ Centrifuge 5430 R, refrigerated microcentrifuge (Fisher Scientific, catalog number: 13-690-005)

9. Water bath (Corning, catalog number: 6783)

10. Pipet-Lite Pipette, Universal SL-1000XLS+ (Mettler Toledo, catalog number: 17014407)

11. Pipet-Lite Pipette, Universal SL-200XLS+ (Mettler Toledo, catalog number: 17014391)

12. Pipet-Lite LTS Pipette L-20XLS+ (Mettler Toledo, catalog number: 17014392)

13. Pipet-Lite LTS Pipette L-10XLS+ (Mettler Toledo, catalog number: 17014388)

14. Pipet-Lite LTS Pipette L-2XLS+ (Mettler Toledo, catalog number: 17014393)

15. Pipet-Lite Pipette Multi L12-1200XLS+ (Mettler Toledo, catalog number: 17014497)

16. Pipet-Lite Multi Pipette L12-300XLS+ (Mettler Toledo, catalog number: 17013811)

17. Pipet-Lite Multi Pipette L12-200XLS+ (Mettler Toledo, catalog number: 17013810)

18. Corning^TM^ 8-channel adapter for vacuum aspirator (Corning, catalog number: 4931)

19. Pipet-Aid^®^ XP (Drummond Scientific Company, catalog number: 4-000-101)

20. Eppendorf^®^ Thermomixer^®^ FP (Millipore Sigma, catalog number: EP5385000024)

21. Vortex Genie-2 (Millipore Sigma, catalog number: Z258423)

22. Forma^TM^ Series II water-jacketed CO_2_ incubator (Thermo Fisher, catalog number: 3131)

23. 1300 Series Class II, Type A2 Biological Safety Cabinet Packages (Thermo Fisher, catalog number: 1323TS)

24. Microscope (Olympus, catalog number: CKX53)

25. Milli Q^®^ IQ 7000 ultrapure water purification system (Millipore Sigma, catalog number: ZIQ7000T0C)

## Software and datasets

1. Fluidigm Software (Standard BioTools, Version 7.1), license required, https://www.fluidigm.com/software

2. Excel (Microsoft, Version 2408), license required, https://www.microsoft.com/en-us/microsoft-365/excel

3. FlowJo (TreeStar, Version 10.10), license required, https://www.flowjo.com/solutions/flowjo/downloads/

4. GraphPad Prism (GraphPad Software LLC., Version 9.5.0), license required, https://www.graphpad.com/scientific-software/prism/

5. RStudio (Posit Software, PBC, Version 2024.09.1+394), no license required, https://posit.co/download/rstudio-desktop/

## Procedure

**A. Stimulation preparation**

*Notes:*

*1. Work in a biological safety cabinet to prepare the 5× stimulation stocks.*

*2. To avoid batch variation, prepare the 5× stimulation stocks for cPBMC and whole blood simultaneously.*

*3. We recommend preparing the 5× stimulation stocks for the cPBMC workflow immediately before use.*

*4. The matrix tubes containing the 5× stimulation stocks can be frozen at -80 °C until required for use.*

*5. Adjust the total volume of 5× stimulation stock based on the number of donors and replicates.*

1. Label sterile 1.5–2.0 mL Eppendorf tubes for each 5× stimulation stock (refer to Table 1).

**Table 1.** Example matrix tube whole blood banking key

Matrix tube color	Catalog number	Stimulation
Clear	Thermo Fisher, 3744	Unstimulated (UNS)
Blue	Thermo Fisher, 3744BLU	IFNα (IFA)
Green	Thermo Fisher, 3744GRN	IFNγ (IFG)
Pink	Thermo Fisher, 3744PIN	Lipopolysaccharide (LPS)
Yellow	Thermo Fisher, 3744YEL	IL-21

2. Prepare 5× stimulation stocks in the associated Eppendorf tubes (refer to Table 2).

**Table 2.** Example of 5× stimulation stock preparation for cPBMC and whole blood

Stimulation	[Stock]	Stimulant [1×]	Stimulant [5×]	5× stimulant (μL)	cRPMI (μL)	Total volume (μL)
IFNα	2.09E+06 IU/mL	10,000 IU/mL	50,000 IU/mL	47.847	1,952.153	2,000.000
IFNγ	25 μg/mL	50 ng/mL	250 ng/mL	20.000	1,980.000	2,000.000
LPS	500 μg/mL	1 μg/mL	5 μg/mL	20.000	1,980.000	2,000.000
IL-21	25 μg/mL	50 ng/mL	250 ng/mL	20.000	1,980.000	2,000.000

Unstimulated samples receive 25 or 50 μL of stimulation media in place of stimuli for cPBMC and whole blood, respectively.

**Figure 2.**
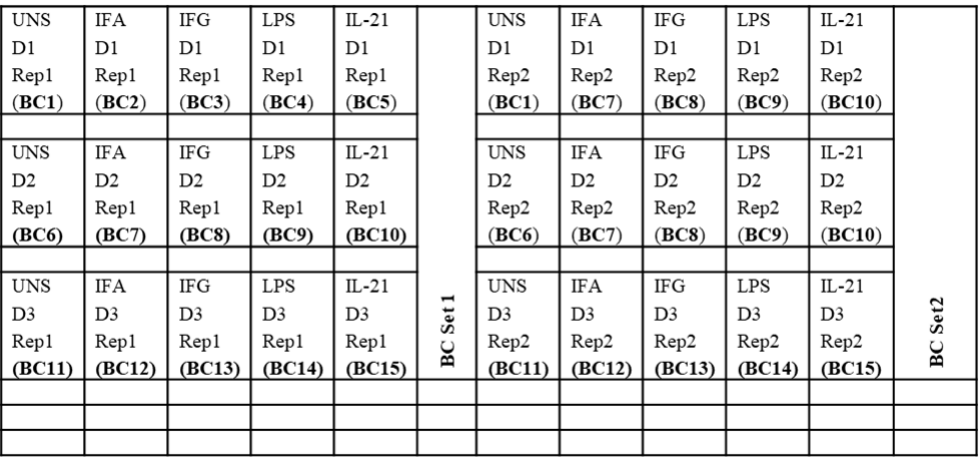
Example universal plate layout (for cPBMC and whole blood processing and barcoding)

3. Aliquot 50 μL of 5× stimulation stock for whole blood into their associated matrix tube (refer to Table 1).

4. Freeze the matrix tubes containing the 5× stimulation stocks at -80 °C until ready for use.

5. Aliquot 25 μL of the corresponding 5× stimulation stocks into each well of a 96-well V-bottom plate (refer to Figure 2) for cPBMC stimulation.

6. Incubate the 96-well plate containing the stimulation at 37 °C in a 5% CO_2_ incubator until the cPBMCs are ready for use.

cPBMCs are transferred directly into the sterile 96-well V-bottom plate containing the 5× stimulation stock (refer to Figure 2).

Whole blood is transferred to a 2 mL deep-well plate for lysis, followed by transferring to a sterile 96-well V-bottom plate for barcoding with the Cell-ID^TM^ 20-Plex Pd Barcoding kit (Section E).

**B. Whole blood stimulation, fixation, and cryopreservation**

*Note: Work in a biological safety cabinet for whole blood stimulation and fixation.*

1. Place whole blood preserved in sodium heparin (green top) collection tubes in a 37 °C, 5% CO_2_ incubator and rest for 30 min to 1 h.

2. Approximately 20 min before use, remove the appropriate number of frozen matrix tubes with the desired stimulants from the -80 °C freezer and thaw at 37 °C in a 5% CO_2_ incubator.

3. Aliquot 200 μL of whole blood (per donor) into the corresponding matrix tubes (refer to Tables 1 and 2).

4. Thoroughly mix the blood and stimulation in each matrix tube.

*Note: Work quickly and change tips between each well to prevent cross-contamination.*

5. Incubate for 15 min at 37 °C in the 5% CO_2_ incubator.

6. Following the same order of whole blood addition, add 250 μL of 1× Proteomic Stabilizer (Prot1) to each matrix tube.

7. Mix thoroughly, changing tips between each sample.

8. Incubate the Prot1 for 10 min at RT.

9. Proceed to Section D for whole blood lysis for immediate sample processing without freezing.

**Pause point:** Stimulated and fixed whole blood can be frozen at -80 °C until it is ready for thawing, processing, and CyTOF analysis.

**C. cPBMC thawing, cisplatin labeling, stimulation, and fixation**

*Notes:*

*1. Work in a biological safety cabinet for cPBMC thawing, cisplatin labeling, stimulation, and fixation.*

*2. Label 14 mL polystyrene round-bottom tubes with donor identification information before thawing the cells.*

*3. Cisplatin labeling (Cell-ID Cisplatin-^195^Pt) is incorporated to assess cell viability post-thawing and prior to processing.*

1. Transfer vials of cPBMCs from liquid nitrogen to dry ice.

2. Thaw cryopreserved PBMCs in a 37 °C water bath for 2 min.

3. Remove cryovials from the water bath and transfer 1 mL of cells into a prelabeled 14 mL polystyrene round-bottom tube.

4. Wash each cryovial with 1 mL of thawing media and transfer to the corresponding 14 mL polystyrene round-bottom tube.

5. Centrifuge at 350× *g* for 5 min at RT.

6. Aspirate the supernatant from each tube.

7. Wash each 14 mL polystyrene round-bottom tube with 10 mL of prewarmed cisplatin labeling media.

8. Centrifuge the Falcon tubes at 350× *g* for 5 min at RT.

9. Use a vacuum aspirator to remove the supernatant from each tube.

10. Resuspend each pellet in 10 mL of prewarmed cisplatin labeling media.

11. Count each 14 mL polystyrene round-bottom tube containing donor PBMCs with an automated cell counter.

12. Centrifuge at 350× *g* for 5 min at RT.

13. Aspirate the supernatant from each tube.

14. Resuspend each cell pellet in cisplatin labeling media at a concentration of 2 × 10^7^ cells/mL.

15. Add an equal volume of 2× Cell-ID Cisplatin-^195^Pt working solution to the resuspended PBMCs (for example, if you are staining 20 million cells in 1 mL, add 1 mL of 2× working solution of ^195^Pt).

16. Mix well and incubate for 5 min at 37 °C in a 5% CO_2_ incubator.

17. Quench the cisplatin labeling reaction with 5 times the volume of prewarmed cRPMI (for example, add 10 mL of cRPMI to 2 mL of a 20 million cell suspension).

18. Centrifuge cells at 350× *g* for 5 min at RT.

19. Aspirate the supernatant from each 14 mL polystyrene round-bottom tube.

20. Add 10 mL of cRPMI and gently mix all samples thoroughly.

21. Centrifuge cells at 300× *g* for 5 min at RT.

22. Aspirate the supernatant and gently pipette to mix.

23. Resuspend cells in 10 mL of cRPMI and rest for 1 h at 37 °C in a 5% CO_2_ incubator.

24. Into a separate 96-well U-bottom plate, distribute 25 μL of each stimulus according to Figure 2 and place it at 37 °C in the 5% CO_2_ incubator.

25. At the end of the rest period, resuspend cells at a total cell concentration of 2 × 10^7^ cells/mL.

26. Transfer 100 μL of cells into the appropriate wells of a 96-well V-bottom plate (refer to Figure 2).

27. Remove plates containing the stimulants from the 37 °C, 5% CO_2_ incubator.

28. Add 25 μL of each stimulant to the corresponding wells in the assay plate.

29. Mix thoroughly by pipetting up and down.

30. Transfer the assay plate into the 37 °C, 5% CO_2_ incubator for 15 min.

31. Remove the assay plate approximately 55 s before the stimulation incubation is complete.

32. Add 20 μL of 16% formaldehyde solution (w/v) (methanol-free) to all samples, locking in their phosphorylation state.

*Notes:*

*1. The final 16% formaldehyde solution (w/v) methanol-free concentration is equivalent to 2.2%.*

*2. The media will turn yellow/orange and then return to its original color after fixation.*

33. Mix thoroughly by pipetting up and down.

34. Incubate the assay plate in the Thermofisher Eppendorf Mixer at 25 °C, shaking at 350 rpm, for 10 min.

35. Add 100 μL of cold 1× BC buffer to each well.

36. Proceed to Section E for barcode preparation and pooling, Section F for barcoding, or Section G for surface staining and methanol permeabilization if samples are not barcoded.

**D. Whole blood lysis**

*Notes:*

*1. If samples were not frozen after stimulation, move immediately to step D2.*

*2. Whole blood lysis can be performed on the bench.*

*3. Do not exceed three lysis steps.*

1. Thaw the matrix tubes by transferring them to 25 °C water for 15 min.

2. Transfer 500 μL of the stimulated and fixed whole blood into a 2 mL deep-well plate containing 1.2 mL of 1× thaw-lyse solution (refer to Figure 2).

3. Mix each well thoroughly with a P1200 multichannel pipette.

4. Allow the whole blood to lyse for 10 min at RT.

5. Centrifuge the deep well containing the lysed whole blood at 548× *g* for 10 min at RT.

6. Carefully remove the supernatant from each well.

7. Seal the top of the deep-well plate with a plate seal and gently vortex the plate.

8. Add 1.6 mL of 1× thaw/lyse and mix thoroughly with a P1200 multichannel pipette.

9. Seal the plate and allow the whole blood to lyse for 10 min at RT.

10. Centrifuge the deep well at 548× *g* for 10 min at RT.

11. Carefully remove the supernatant from each well.

12. Seal the top of the deep well plate with a plastic plate seal and gently vortex the plate.

13. If substantial red blood cells (RBCs) remain, repeat an additional lysis (steps D8–11). If not, wash the pellet with 1.6 mL of cell staining buffer (CSB).

14. Centrifuge cells at 974× *g* for 10 min at 4 °C.

15. Carefully remove the supernatant, leaving approximately 50 μL of volume at the bottom of each well.

16. Resuspend each well with an additional 100 μL of CSB.

17. Transfer the cell suspension into the corresponding wells of a 96-well V-bottom plate (refer to Figure 2).

18. Centrifuge the 96-well V-bottom plate containing the cell suspensions at 974× *g* for 5 min at 4 °C.

19. Carefully remove the supernatant.

20. If any liquid remains in the deep-well plate from step D17, transfer the remaining volume into the corresponding wells of the 96-well V-bottom plate.

21. Proceed to Section E for barcode preparation and pooling, Section F for barcoding, or Section G for surface staining and methanol permeabilization.

**E. Barcode preparation and pooling (optional)**

*Notes:*

*1. Barcodes for preserved whole blood samples can be prepared the day before processing on the bench.*

*2. A single barcode ampule is sufficient for two wells of whole blood or three wells of cPBMCs.*

1. Remove the appropriate barcode ampules from the Cell-ID^TM^ 20-Plex Pd Barcoding kit stored at -20 °C and place them on wet ice to thaw.

2. Based on the plate layout (see Figure 2), remove enough ampules to provide 30 μL of barcodes per well for cPBMCs or 50 μL of barcodes per well for whole blood.

3. Resuspend each ampule of barcodes with 100 μL of 1× BC buffer.

4. Transfer the contents of each barcode ampule into a single well of a 96-well U-bottom plate, ensuring that you do not exceed two ampules of the same barcode in any one well.

5. Keep the 96-well U-bottom plate containing the resuspended barcodes at 4 °C until ready for use.

**F. Barcoding (optional)**

*Note: Barcoding for both whole blood and cPBMC can be performed on the bench.*

1. Centrifuge the 96-well V-bottom plate at 974× *g* for 5 min at 4 °C.

2. Flick the supernatant from the plate and blot on a clean paper towel to remove residual liquid.

3. Resuspend cells in 200 μL of ice-cold 1× BC buffer and centrifuge at 974× *g* for 5 min at 4 °C.

4. Flick the supernatant from the plate and blot the plate on a clean paper towel to remove residual liquid.

5. Resuspend each well in 50 μL of the appropriate barcode for whole blood or 30 μL for cPBMCs.

6. Incubate the 96-well V-bottom plate on the thermomixer at 25 °C, shaking at 350 rpm, for 30 min.

7. Add enough ice-cold 1× BC buffer to all wells to bring the total volume to 200 μL/well.

8. Centrifuge the 96-well V-bottom at 974× *g* for 5 min at 4 °C.

9. Flick the supernatant from the plate and blot on a clean paper towel to remove residual liquid.

10. Resuspend all wells in 100 μL of CSB and combine up to 20 barcoded samples into a 2 mL Eppendorf tube (refer to Figure 2; for example, BC Set 1 and 2 contain BCs 1–15, which are pooled into two Eppendorf tubes).

11. Rinse each well with 100 μL of CSB and combine the contents into a second set of 2 mL Eppendorf tubes.

12. Centrifuge all tubes in the microcentrifuge at 1,000× *g* for 5 min at 4 °C.

13. Carefully aspirate the supernatant.

14. Pool the primary and wash tubes by resuspending each in 500 μL of CSB and transferring the contents of the wash tube into the primary tube.

15. Raise the volume of the primary Eppendorf tube to 2 mL with CSB.

16. Centrifuge all tubes in the microfuge at 1,000× *g* for 5 min at 4 °C.

17. Carefully aspirate the supernatant.

18. Measure any residual volume and correct it to a volume equating to 50 μL per barcoded sample.

**G. Surface staining and methanol permeabilization**

*Note: Surface staining and methanol permeabilization can be performed on the bench.*

1. Prepare the 2× surface master mix (refer to Table 3).

**Table 3.** Surface master mix for immunophenotyping of human samples (example for 60 samples) *Whole blood panel only. #The purified antibody was metal labeled in-house for CyTOF use.

Metal	Target	Vendor	Catalog number	Clone	Ab volume (μL)
^141^Pr	CD7#	BioLegend	343111	CD7-6B7	8.75
^151^Eu	CD123 (IL-3R)	Standard BioTools	3151001B	6H6	8.75
^155^Gd	CD27	Standard BioTools	3155001B	L128	8.75
^159^Tb	CD11c	Standard BioTools	3159001B	Bu15	8.75
^176^Yb	CD127 (IL-7Ra)	Standard BioTools	3176004B	A019D5	17.5
^142^Nd	CD19	Standard BioTools	3142001B	HIB19	17.5
^167^Er	CD38	Standard BioTools	3167001B	HIT2	17.5
^170^Er	CD3	Standard BioTools	3170001B	UCHT1	17.5
^174^Yb	HLA-DR	Standard BioTools	3174001B	L243	17.5
^162^Dy*	CD66b*	Standard BioTools*	3162023B*	80H3*	35*
^112^Cd	CD8a	Standard BioTools	92J035112	SK1	35
^209^Bi	CD16	Standard BioTools	3209002B	3G8	35
^145^Nd	CD4	Standard BioTools	3145001B	RPA-T4	35
^160^Gd	CD14	Standard BioTools	3160006B	RMO52	35
^147^Sm	CD20	Standard BioTools	3147001B	2H7	70
^89^Y	CD45	Standard BioTools	3089003B	HI30	43.75
^146^Nd	IgD	Standard BioTools	3146005B	IA6-2	43.75
^149^Sm	CD25 (IL-2R)	Standard BioTools	3149010B	2A3	43.75
^163^Dy	CD56	Standard BioTools	3163007B	NCAM16.2	21.875
^169^Tm	CD45RA	Standard BioTools	3169008B	HI100	10.938
Volume of cell staining buffer (CSB)					3003.44

2. Add 50 μL of 2× surface master mix per barcoded sample to the 2 mL Eppendorf tubes.

3. Incubate the 2 mL Eppendorf tubes on the thermomixer for 30 min at 25 °C with a shaking speed of 350 rpm.

4. Centrifuge the 2 mL Eppendorf tubes at 1,000× *g* for 5 min at 4 °C.

5. Carefully aspirate the supernatant.

6. Resuspend the 2 mL Eppendorf tubes with 1.8 mL of CSB.

7. Centrifuge at 1,000× *g* for 5 min at 4 °C.

8. Carefully aspirate the supernatant.

9. Repeat steps G6–8 once more.

10. Add 800 μL of ice-cold 100% MeOH dropwise.

11. Add an additional 1 mL of 100% MeOH dropwise to each 2 mL Eppendorf tube and incubate on ice for 30 min.

**Pause point:** Samples can be stored at -80 °C overnight in 100% MeOH to facilitate the workflow. If required, samples can be stored for 4–6 weeks in MeOH.

*Note: If samples require intracellular staining, proceed to Section H.*

**H. Intracellular staining and iridium intercalation**

*Note: Intracellular staining and iridium intercalation can be performed on the bench.*

1. Remove the Eppendorf tubes from the -80 °C freezer and transfer them to wet ice for 30 min.

2. Centrifuge at 1,000× *g* for 10 min at 4 °C.

3. Carefully aspirate the supernatant.

4. Resuspend each 2 mL Eppendorf in 1.8 mL of CSB.

5. Centrifuge at 1,000× *g* for 10 min at 4 °C.

6. Carefully aspirate the supernatant.

7. Repeat steps H4–6 one more time.

8. Measure the residual volume in each 2 mL Eppendorf.

9. If the volume is less than 50 μL per barcode sample, add enough CSB to ensure the final volume is equivalent to # barcodes × 50 – residual volume) (i.e., 750 μL – residual volume for 15 barcodes).

10. Prepare the 2× intracellular master mix (refer to Table 4).

**Table 4.** Intracellular master mix for phospho-analysis of stimulated human samples (example for 60 samples)

Metal	Target	Vendor	Catalog number	Clone	Ab volume (μL)
^144^Nd	pPLCg2 (pY759)	Standard BioTools	3144015A	K86-689.37	8.75
^165^Ho	pCREB (S133)	Standard BioTools	3165034D	87G3	8.75
^175^Lu	pS6 (S235/S236)	Standard BioTools	3175009A	N7-548	8.75
^156^Gd	pP38 [T180/Y182]	Standard BioTools	3156002A	D3F9	17.5
^171^Yb	pERK 1/2 [T202/Y204]	Standard BioTools	3171010A	D13.14.4E	17.5
^152^Sm	pAkt (S473)	Standard BioTools	3152005A	D9E	35
^153^Eu	pSTAT1 [Y701]	Standard BioTools	3153003A	58D6	35
^158^Gd	pSTAT3 [Y705]	Standard BioTools	3158005A	4/P-STAT3	35
^148^Nd	pSTAT4 [Y693]	Standard BioTools	3148006A	38/p-Stat4	35
^150^Nd	pSTAT5 [Y694]	Standard BioTools	3150005A	47	35
^168^Er	pSTAT6 [Y641]	Standard BioTools	3168012A	18/P-Stat6	35
^164^Dy	IKβα	Standard BioTools	3164004A	L35A5	35
Volume of cell staining buffer (CSB)					3193.75

11. Add a volume of 2× intracellular master mix equating to 50 μL per barcoded sample to each 2 mL Eppendorf tube.

12. Incubate the Eppendorf tube in the thermomixer at 25 °C, shaking at 350 rpm for 30 min.

13. Centrifuge at 1,000× *g* for 10 min at 4 °C.

14. Carefully aspirate supernatant.

15. Resuspend each 2 mL Eppendorf tube in 1.8 mL of CSB.

16. Centrifuge at 1,000× *g* for 5 min at 4 °C.

17. Carefully aspirate supernatant.

18. Repeat steps H14–16 one more time.

19. Resuspend each 2 mL Eppendorf tube in 100 μL of iridium intercalation solution per barcoded sample and store at 4 °C for a minimum of 30 min.

**Pause point:** Samples can be stored at 4 °C in iridium intercalation solution overnight. Do not exceed this timing.

**I. Sample preparation for Helios acquisition**

*Note: Sample preparation for acquisition on the CyTOF can be performed on the bench.*

1. Start up the Helios (total duration ~1 h):

a. Start-up plasma (during this time, process samples as indicated below) (duration ~20 min).

b. Install nebulizer and check nebulizer spray (duration ~2–5 min).

c. Check background (duration ~1–2 min).

d. Tune (duration ~16 min).

e. Run EQ4 beads (duration ~2 min).

f. Run CAS^+^ buffer for a minimum of 15 min to precondition the wide-bore injector.

2. Retrieve samples from the 4 °C and mix by vortexing.

3. Add enough CSB to each 2 mL Eppendorf tube to bring the total volume to 1.8 mL.

4. Centrifuge at 1,000× *g* for 5 min at 4 °C.

5. Carefully aspirate the supernatant without dislodging the pellet.

6. Resuspend cells in 1.8 mL of CSB.

7. Centrifuge at 1,000× *g* for 5 min at 4 °C.

8. Carefully aspirate the supernatant without dislodging the pellet.

9. Repeat steps I6–8 once more.

**Pause point:** Aspirate all but 50 μL of CSB from each tube.

*Notes:*

*1. Process each Eppendorf Tube one at a time.*

*2. When all samples cannot be run on the same day, centrifuge and remove all but 50 μL of CSB from the 2 mL Eppendorf tube. Store samples at 4 °C, sealed with parafilm to prevent evaporation.*

*3. These samples should be run as soon as possible, preferably the next day.*

**Caution:** Only samples that can be run in one day should be processed from CSB into CAS^+^ to avoid cell loss.

10. Resuspend cells in 1.8 mL of CAS^+^.

11. Centrifuge at 1,000× *g* for 10 min at 4 °C.

12. Carefully aspirate the supernatant without dislodging the pellet.

13. Resuspend cells in 1.8 mL of CAS^+^.

14. Repeat steps I10–12 once more.

15. Resuspend cells in 1 mL of freshly prepared 1:10 EQ4 beads diluted in CAS^+^.

16. Place the samples on ice.

17. Remove 10 μL for counting using a C-chip hemocytometer on the microscope.

18. Count the cells and adjust the concentration to 1.3 × 10^6^ cells/mL in 1:10 EQ4 beads diluted in CAS^+^. Aim to achieve an event rate of ~350–400 events/s (± 50 events/s).

*Note: The final volume for resuspension is dependent on the cell count from step I18*

19. Proceed to run cells on the Helios mass cytometer.

## Data analysis

Following acquisition on Helios, datasets undergo standard preprocessing for downstream analyses. The intricate details of CyTOF data analyses are beyond the scope of this protocol; however, they have been thoroughly reviewed in other protocols [16]. In summary, the data undergoes a preprocessing step to ensure the quality and clarity of the data. Raw .fcs files generated after sample acquisition undergo processes of normalization and randomization using the CyTOF Software v.7.1 (Standard BioTools). Inclusion of the EQ Four Element beads (containing isotopes of cerium, europium, holmium, and lutetium) is critical for data normalization. The CyTOF software normalizes the data to a global bead standard and corrects for signal fluctuations or saturation occurring during data collection. This process ensures that data collected at different times or across different runs are comparable, making this step vital for consistency. Data randomization smooths the raw data to improve its overall quality. Following this, additional essential preprocessing steps are performed to refine the dataset, including the identification of real nucleated cell events using the Cell-ID^TM^ intercalation solution. Viability gating through the use of cisplatin permits the exclusion of dead or dying cells. De-barcoding allows for the separation of multiplexed samples into individual .fcs files for each sample. These post-normalization steps are executed using FlowJo (TreeStar, Inc) or by leveraging publicly available R packages [17].

**Figure 3.**
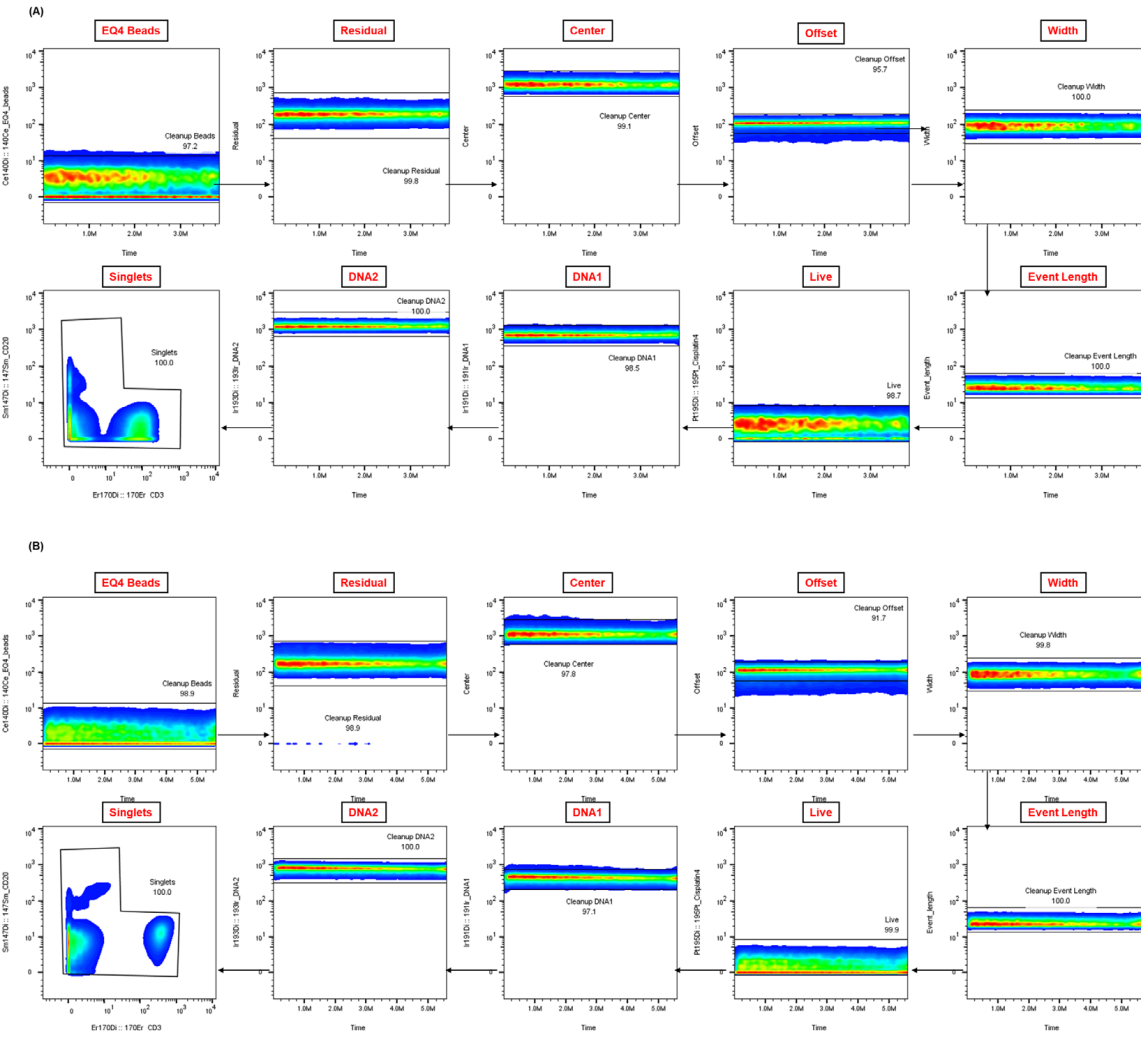
Representative Gaussian parameter cleanup for cryopreserved peripheral blood mononuclear cells (cPBMCs) and whole blood CyTOF data. The application of Gaussian parameter cleanup to raw mass cytometry data is an essential step in enhancing data quality. Shown are representative examples from matched (**A**) cPBMCs and (**B**) fresh whole blood (WB), both from a single healthy donor. This step is crucial for isolating real cellular events and enhancing the resolution of subsequent analysis.

**Figure 4.**
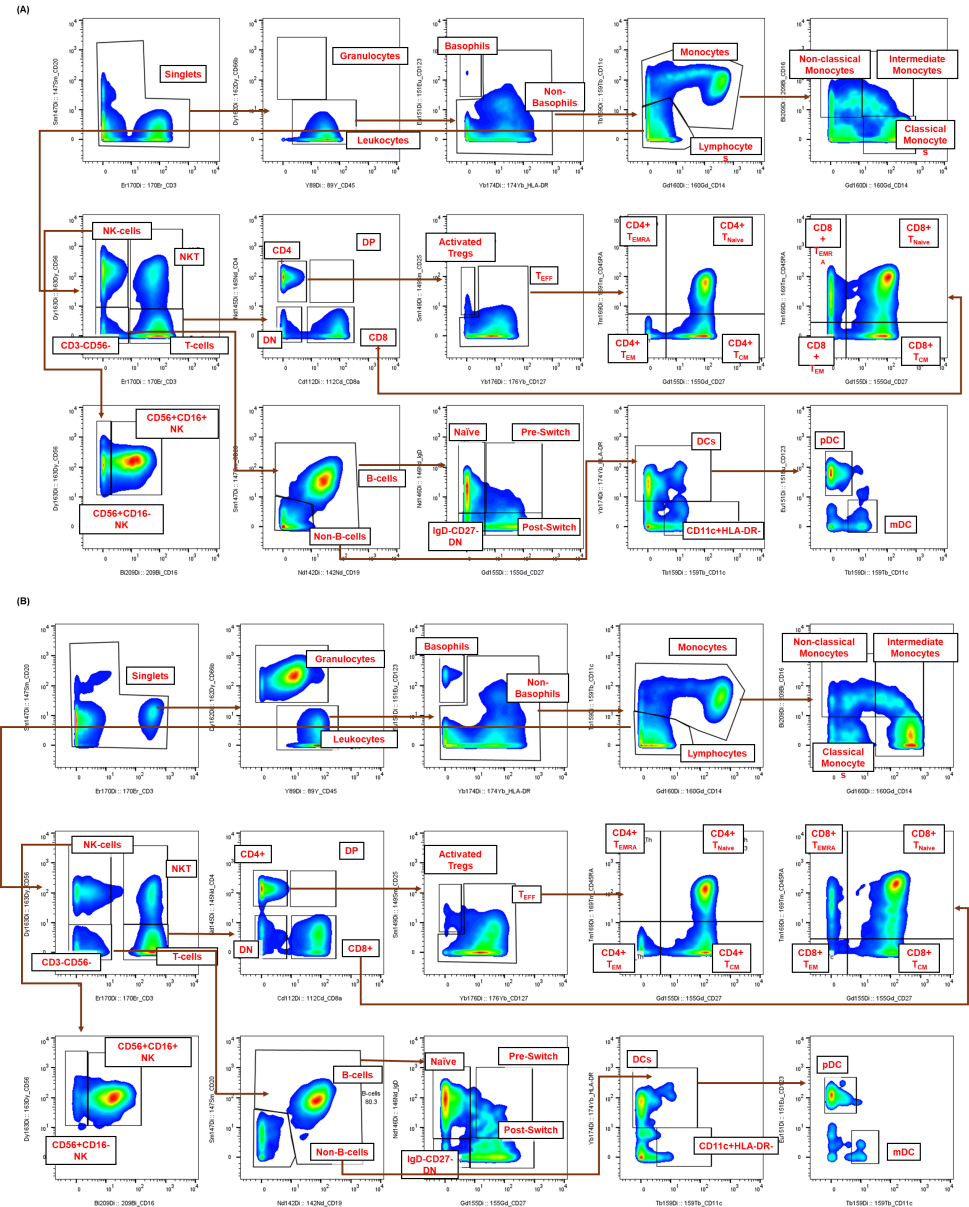
Representative gating strategy for high-dimensional immunophenotyping of healthy human immune subsets by CyTOF. The sequential gating strategy was used to identify immune cell populations from healthy human samples. Healthy donor samples, either (**A**) cryopreserved peripheral blood mononuclear cells (cPBMCs) or (**B**) fresh whole blood (WB), were stimulated for 15 min, fixed, palladium-barcoded, and stained with a comprehensive panel of 19 (for cPBMCs) or 20 (for whole blood) surface markers. Within the single-cell gate, non-granulocytes (leukocytes) were defined as CD66b-CD45^+^, while granulocytes were confirmed solely in whole blood as CD66b^+^CD45-. Lymphocytes were resolved using CD3 and CD56 [T cells (CD3^+^CD56-)], NKT cells (CD3^+^CD56^+^), and NK cells (CD3-CD56^+^). B cells were identified as CD19^+^CD20^+/-^ within the CD3-CD56- gate, further confirmed by negative expression of CD123 and CD11c. Both T and B cells were further subset based on the expression of CD4, CD8, CD45RA, CD27, and CD25 or IgD and CD27, respectively. Non-T, non-B, and non-NK cells (CD45^+^CD3-CD19-CD56-CD20-) were separated based on their expression of CD11c and HLA-DR. Total DCs were defined as CD11c^+^HLA-DR^+^ and can be further defined through the expression of CD123 (plasmacytoid DCs). Monocytes were resolved within this non-lymphocyte gate based on their expression of CD14 and CD16.

**Figure 5.**
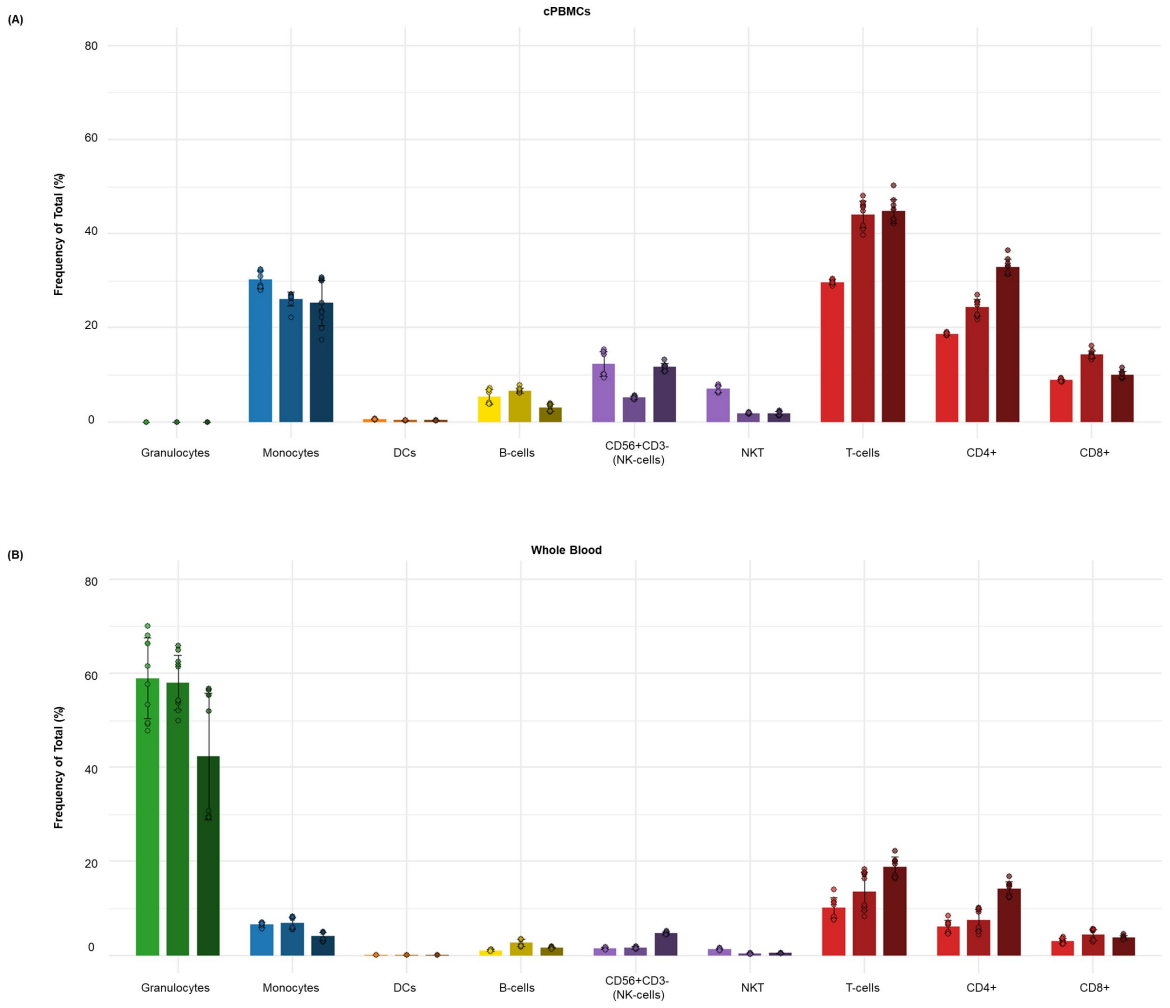
Distribution of major immune subsets in healthy human donors by dual workflow phospho-CyTOF. This bar graph illustrates the average frequency of total (%) of the major immune cell subsets in donor-matched (**A**) cPBMCs and (**B**) whole blood from (n = 3) healthy individuals. The data points represent the average frequency of total (%) of each subset, with error bars indicating the standard deviation from triplicate samples. Major immune subsets, including B cells, monocytes, dendritic cells (DCs), NK cells, NKT cells, and T cells, were identified using a comprehensive high-dimensional immunophenotyping strategy defined after Gaussian parameter cleanup (Figure 2) and doublet exclusion. These lineages were identified through expression of markers such as CD3, CD19, CD56, CD14, CD11c, HLA-DR, and CD66b, following the detailed gating strategy outlined in Figure 3. As expected, cPBMCs have a higher proportional representation of lymphocytes (T cells, B cells, NK, and NKT cells) and monocytes but lack granulocytes, whereas whole blood maintains the complete cellular repertoire, including a larger proportion of granulocytes and what appears to be a lower percentage of lymphocyte or monocyte subsets due to being expressed as a percentage of total leukocytes.

**Figure 6.**
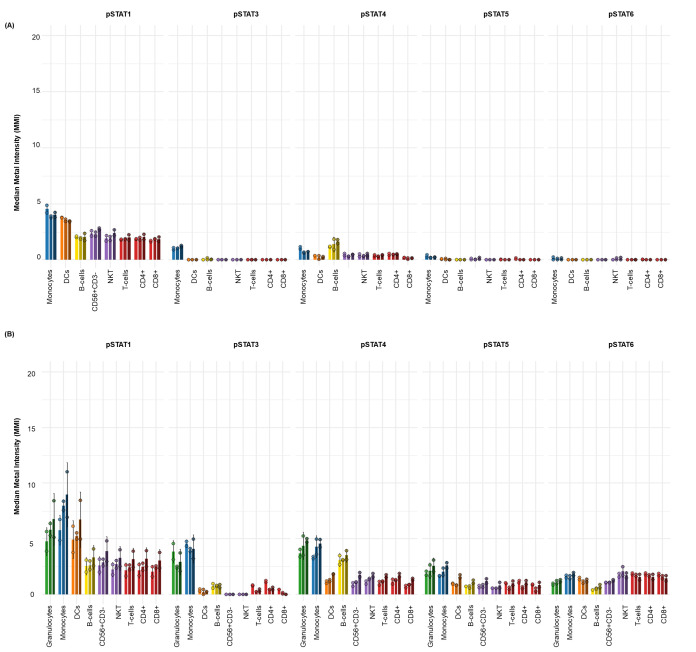
Elevated basal phospho-STAT levels in whole blood compared to cryopreserved peripheral blood mononuclear cells (cPBMCs). The baseline (unstimulated) median metal intensity (MMI) of phospho-STAT molecules (pSTAT1, pSTAT3, pSTAT4, pSTAT5, and pSTAT6) in matched samples from three healthy donors (n = 3). The bar graphs illustrate basal phospho-STAT levels in (**A**) cPBMCs and (**B**) whole blood (WB). Data are presented as the mean MMI across three healthy donors (n=3), with error bars indicating the standard deviation of two technical replicates. Notably, whole blood consistently exhibits higher basal phospho-STAT levels across all measured STATs compared to cPBMCs. Despite these differences in magnitude, both cPBMC and whole blood samples show a similar qualitative pattern of increased basal pSTAT1, pSTAT3, and pSTAT4. In contrast, basal pSTAT5 and pSTAT6 levels are generally lower in cPBMCs than in whole blood, consistent with their typically low basal expression.

**Figure 7.**
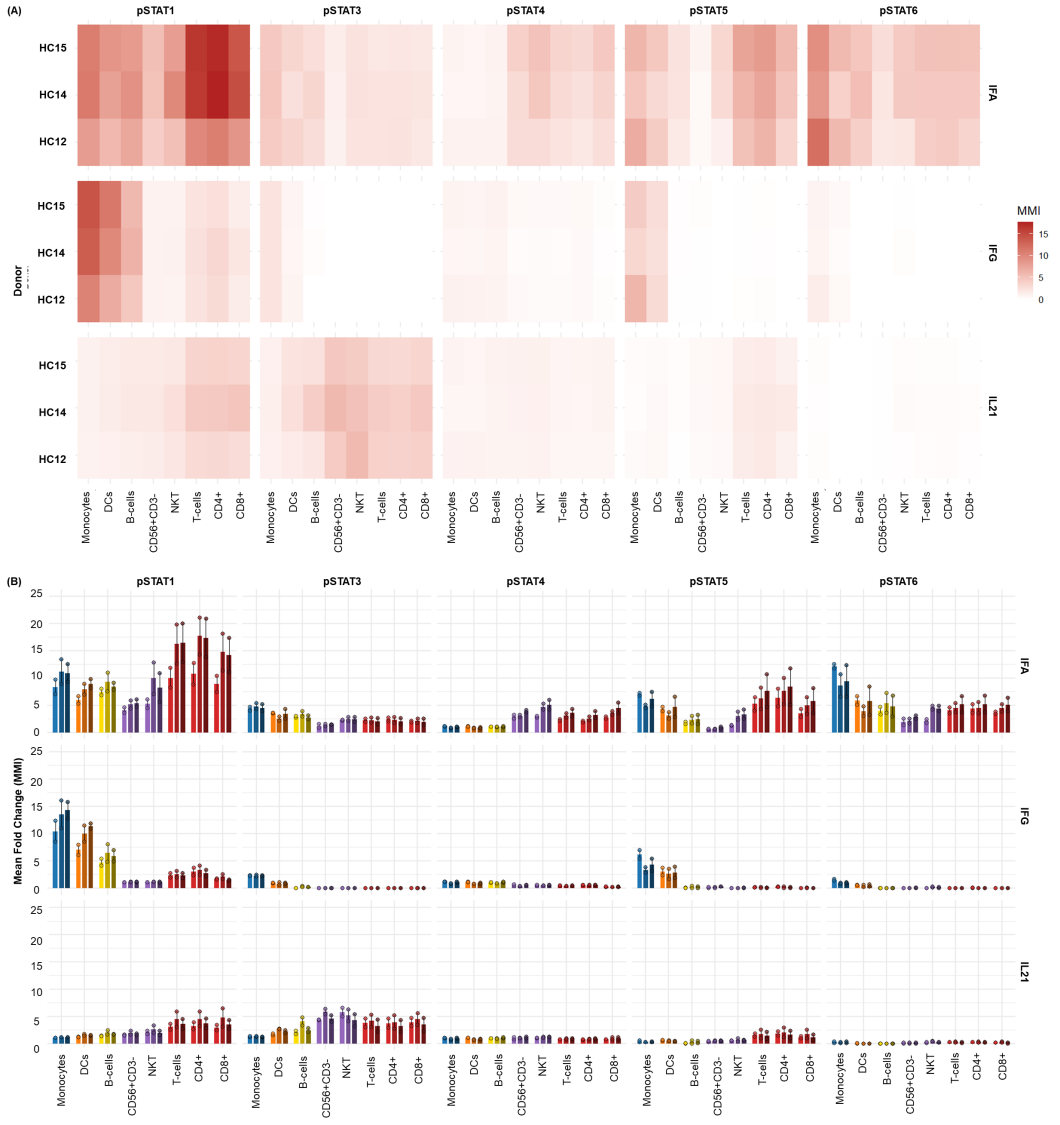
Differential JAK/STAT phosphorylation in healthy cryopreserved peripheral blood mononuclear cells (cPBMCs) following cytokine stimulation. The distinct phospho-STAT responses across major immune cell subsets found in (n = 3) healthy donor cPBMCs with two technical replicates per donor after 15 min of stimulation with IFNα (1e4 U/mL), IFNγ (50 ng/mL), and IL-21 (50 ng/mL). (**A**) Heatmaps display the fold change in phosphorylation for STAT1, STAT3, STAT4, STAT5, and STAT6 across major immune subsets. Fold change for each intracellular cell-state marker was calculated using the formula MMI_stim_/MMI_unstim _for each donor and displayed as the average of duplicates. (**B**) Bar graphs illustrating the average fold change over unstimulated (mean ± SD) in phosphorylation for each STAT protein within key immune cell populations [B cells, Monocytes, dCs, NK cells, NKT cells, and T cells (CD4^+^ and CD8^+^)] across three donors.

**Figure 8.**
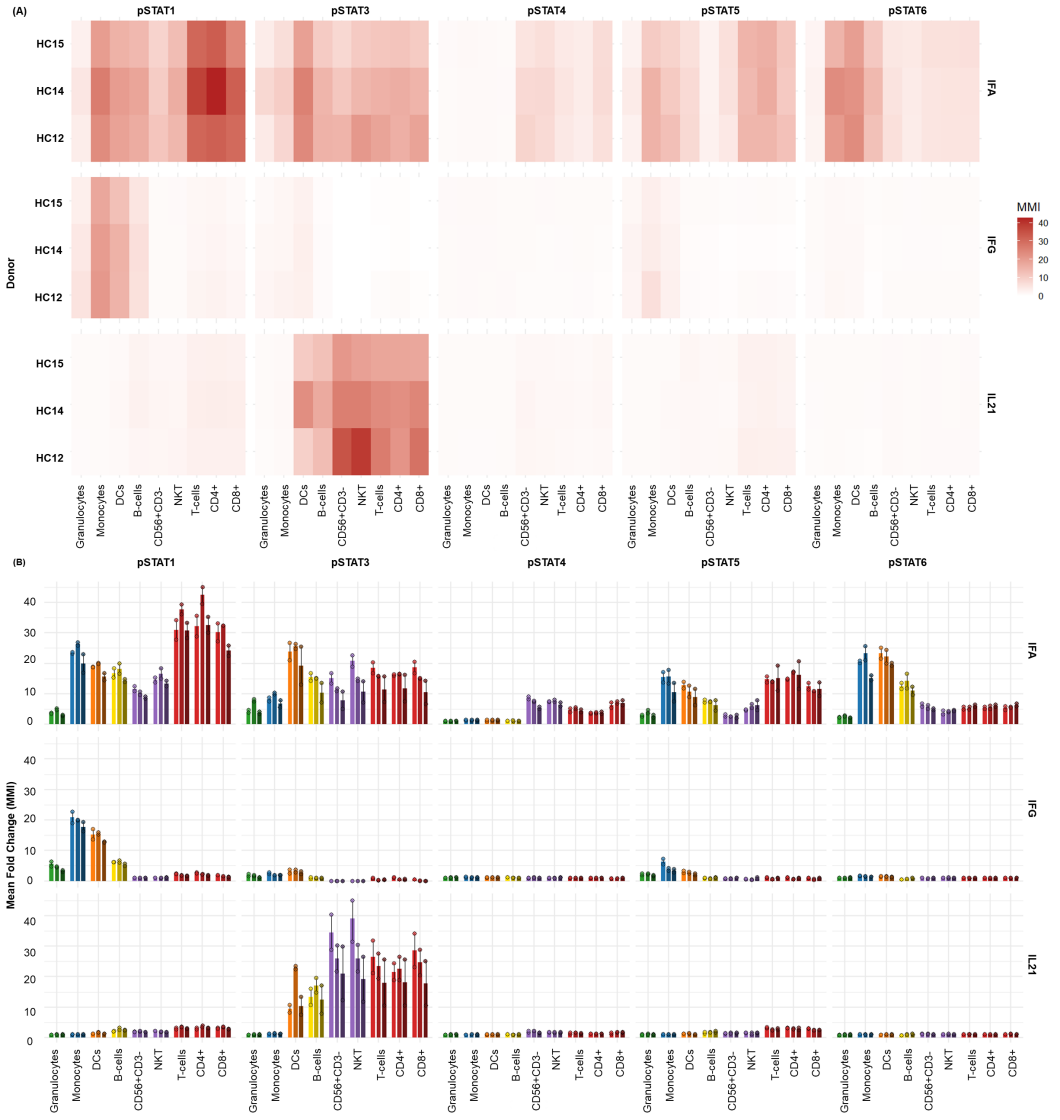
Whole blood exhibits distinct STAT phosphorylation profiles upon type I/II Interferon and IL-21 stimulation. The distinct phospho-STAT responses across major immune cell subsets found in donor-matched (n = 3) whole blood with two technical replicates per donor after 15 min of stimulation with IFNα (1e4 U/mL), IFNγ (50 ng/mL), and IL-21 (50 ng/mL). (**A**) Heatmaps display the fold change in phosphorylation for STAT1, STAT3, STAT4, STAT5, and STAT6 across major immune subsets. Fold change for each intracellular cell-state marker was calculated using the formula MMI_stim_/MMI_unstim_ for each donor and displayed as the average. (**B**) Bar graphs illustrating the average fold change over unstimulated (mean ± standard deviation) in phosphorylation for each STAT protein within key immune cell populations [granulocytes, monocytes, DCs, B cells, NK cells, NKT cells, and T cells (CD4^+^ and CD8^+^)] across all three donors.

After these datasets are preprocessed and cleaned via Gaussian parameter clean-up (outlined in Figure 3) using FlowJo software (TreeStar, Inc.), a comprehensive, supervised gating strategy (refer to Figure 4) is employed to identify and quantify the frequencies of immune cell subsets (e.g., T cells, B cells, monocytes, DCs, NK cells, NKT cells). The results are reported as the relative frequency of total (FT%) and displayed as bar graphs representing the average of two technical replicates for three biological replicates, along with the standard deviation to illustrate trends in the data (refer to Figure 5 for abridged immunophenotyping and Figure S1 for the full immunophenotyping results). Baseline quantification of protein phosphorylation is achieved using the unstimulated sample’s median metal intensity (MMI) for each intracellular epitope on all relevant cell populations (refer to Figure 6). To determine each immune subset’s response to stimulation for all intracellular epitopes, the negative raw MMI values are corrected to zero. For unstimulated samples, all MMI values of zero are adjusted to one to establish a baseline. To assess differences in phosphorylation status, we manually calculate the fold change over unstimulated for each phospho-protein using the formula MMI_stim_/MMI_unstim_ for each donor and display the resultant value as heatmaps (refer to Figure 7A and 8A) with increased phosphorylation represented as darker red for that population–stimulation combination or as the average of duplicates in bar graphs (refer to Figure 7B and 8B). This approach allows us to determine increases in phosphorylation for each donor. The calculated fold change in MMI can be plotted with GraphPad Prism to illustrate differences in the magnitude of the phosphorylation responses across various cell population–stimulation combinations (i.e., pSTAT molecules). All downstream analysis was performed using RStudio to gain deeper insight into differences in immune composition and donor-specific phospho-responses to IFNα, IFNγ, IL-21, and LPS.

**Quantification and statistical analysis**

All experiments were performed with three independent healthy donors as biological replicates. For each healthy donor, duplicate technical measurements were obtained as described below in Validation of Protocol. The frequency of total (%) for each cell population was determined by supervised manual gating using FlowJo. Data analyses were performed using the mean of duplicate technical replicates for each of the three independent biological donors using R software (version 4.2.0) or GraphPad Prism 9.0. Due to the limited number of biological replicates, formal statistical significance testing was not performed. Instead, descriptive statistics (mean ± standard deviation) are reported to illustrate trends across donors. Where applicable, differences between conditions are visually represented and annotated in figure legends. These data are intended to support biological interpretation rather than infer population-level significance. However, when there are sufficient technical replicates to compare two groups (e.g., control vs. stimulated), an unpaired two-tailed Student’s t-test can be used. If data is reflective of three or more groups, a one-way analysis of variance (ANOVA) followed by multiple comparisons (e.g., Tukey's post-hoc test) can be employed to identify statistically significant differences between specific group pairs.

An internally developed R script was employed to convert the FlowJo export tables into graphics [18]. Written for R 4.3 in RStudio [19], the script (i) ingests the worksheets generated at the gating step, (ii) harmonizes donor, replicate, and stimulation metadata, and (iii) reshapes the frequency and median-intensity matrices into a tidy long format with readxl, dplyr, tidyr, and stringr [20–22]. Plotting is performed with ggplot2 [23], while color management relies on RColorBrewer, viridis, and scales [24–26] to ensure palette consistency across figures. The same script then (a) summarizes relative immune-subset frequencies (mean ± SD or 0%–100% normalized compositions), (b) visualizes baseline median metal intensities, and (c) displays stimulation-induced fold-changes, and exports all figures automatically in both raster (JPEG) and vector (PDF) formats.

## Validation of protocol

Dual workflow implementation required rigorous validation of stimulation conditions, fixation protocols, and titration of newly integrated antibody-metal conjugates (CD66b ^162^Dy, CD8a ^112^Cd, pSTAT4 ^148^Nd, pSTAT6 ^168^Er; Figure S2). Protocols for PBMC and whole blood stimulation were adapted from established *Bio-Protocol* methods by Fernandez and Maecker (2015) for cytokine-stimulated phosphoflow [13,15].

The Jurkat (T-cell proxy), Raji (B-cell proxy), and THP-1 (monocyte proxy) cell lines were used to determine the optimal duration of IFNα stimulation. A 15-min stimulation consistently induced phosphorylation of STAT1, STAT3, and STAT5 (refer to Figure S3), and was selected for all subsequent assays. To expand phospho-epitope coverage within the CyTOF mass window, antibody-metal conjugates were titrated on cPBMCs and whole blood. CD8a ^112^Cd and CD66b ^162^Dy were optimized at a 1:200 dilution. pSTAT4 ^148^Nd was titrated in response to IL-4 stimulation, with 1:200 selected for downstream use. pSTAT6 ^168^Er was validated on cPBMCs (refer to Figure S2). Following optimization, cPBMCs from two healthy donors were used to validate the surface (refer to Table 3) and intracellular master mix (refer to Table 4) panels. Cytokine-specific STAT phosphorylation patterns were confirmed in CD4^+^ T cells, B cells, and monocytes (refer to Figure S4):

• **CD4^+^ T cells:** pSTAT1 induced by IFNα/γ; pSTAT3 by IL-6/IL-21; pSTAT4 by IL-21; pSTAT6 by IL-4.

• **B cells:** pSTAT1 by IFNα/γ; pSTAT3 by IL-6/IL-21; pSTAT4 by IFNγ/IL-21; pSTAT6 by IL-4.

• **Monocytes:** pSTAT1 by IFNα/γ; pSTAT3 by all cytokines; pSTAT4 by IFNγ/IL-21; pSTAT6 by IL-4.

These results aligned with known STAT activation profiles (refer to Table S3) and confirmed panel sensitivity.

Validated conditions were applied to profile three healthy donors (two technical replicates each) to assess reproducibility. The surface master mix panel (refer to Table 3) resolved 39 immune subsets in cPBMCs and whole blood (refer to Figure S1). The intracellular master mix panel (refer to Table 4) profiled stimulation responses to IFNα, IFNγ, IL-21, and LPS (refer to Figures 7, 8, S5, S6, and S7 and Tables S3 and S4). Unstimulated controls enabled baseline normalization and fold-change calculations in median metal intensity (MMI). Cell-ID^TM^ iridium intercalator was used for doublet exclusion; cisplatin labeling was used solely in cPBMCs to assess viability post-thaw.

**cPBMCs:**

• IFNα induced pan-STAT phosphorylation (STAT1 > STAT6 > STAT5 > STAT4 ≈ STAT3).

• IFNγ triggered focused STAT1 activation in APCs and moderate STAT3/5 in monocytes/DCs.

• IL-21 activated STAT3 broadly (NK, NKT, T, B, DCs) and STAT1/5 in T/NKT cells.

**Whole blood:**

• IFNα induced broad STAT activation, with the strongest response in STAT1 and STAT3 across various immune subsets.

• IFNγ primarily activated STAT1 in monocytes/DCs; modest STAT5 activation was observed in monocytes.

• IL-21 selectively induced STAT3 in both lymphoid and myeloid subsets, with minimal activation of other STATs.

LPS stimulation served as a myeloid-specific control, inducing p38, ERK1/2 phosphorylation, and IκBα degradation (refer to Table S4 and Figure S7) in both cPBMCs and whole blood, with minimal or no significant changes observed in the lymphocyte populations (i.e., B cells, CD4^+^ T cells, CD8^+^ T cells, NK cells, NKT cells). The data presented confirms the expected activation of the MAPK pathway (pERK1/2, p38) and NFκB pathway (IκBα degradation) in response to LPS stimulation, predominantly in myeloid cell subsets, in both cryopreserved PBMCs and whole blood. Differences in the magnitude of response between cPBMCs and whole blood are consistent with the distinct cellular compositions and environmental factors of these sample types. All datasets were benchmarked against prior data generated in-lab and published literature to confirm expected cytokine-induced STAT phosphorylation patterns (refer to Figures S6 and S7).

The choice between whole blood and cPBMCs depends on the scientific question and technical constraints, primarily due to differences in cellular composition. Whole blood retains granulocytes (neutrophils, eosinophils, basophils), preserving in vivo cell–cell interactions lost during PBMC isolation (refer to Figure 5B). In contrast, cPBMCs are enriched for mononuclear cells (lymphocytes, monocytes), enabling focused analysis of specific subsets but lacking systemic context (refer to Figure 5A). Basal phospho-STAT levels differ between sample types, with whole blood consistently showing higher baseline and stimulated responses, particularly for myeloid-targeted stimuli (refer to Figure 6). This may reflect stress-induced activation during PBMC isolation and cryopreservation or a more quiescent post-thaw state (refer to Figure 6A). Whole blood also exhibits greater absolute phospho-STAT magnitudes, potentially due to granulocyte contributions or intact signaling environments (refer to Figure 6B).

Comparative analysis of IFNα stimulation revealed broad pSTAT1 induction across T cells, B cells, monocytes, and NK cells in both sample types, with higher magnitude in whole blood. Hierarchies of STAT activation differed slightly: STAT1 > STAT3 > STAT6 > STAT5 > STAT4 in whole blood vs. STAT1 > STAT6 > STAT5 > STAT4 > STAT3 in cPBMCs (refer to Figures 7 and 8 and Table S3). IFNγ stimulation produced strong pSTAT1 responses in monocytes, DCs, and B cells, with lesser activation in T cells. Whole blood again showed higher magnitude, especially in myeloid subsets, possibly due to preserved signaling factors or reduced isolation stress. Both sample types showed minimal pSTAT3 and pSTAT5 induction with IFNγ, suggesting low-level crosstalk. These responses were more consistently detectable in whole blood. A notable divergence emerged with IL-21 stimulation. While both sample types showed robust pSTAT3 activation in T cells, B cells, NK cells, and NKT cells, cPBMCs exhibited co-activation of STAT1 and STAT5 alongside STAT3 (refer to Figure 7). In contrast, whole blood displayed a more specific STAT3 response with minimal STAT1/5 induction (refer to Figure 8). This may reflect modulatory effects of granulocytes or cytokine competition in whole blood.

This validation confirms that the protocol reliably detects cytokine-specific STAT phosphorylation across diverse immune subsets in both cPBMCs and whole blood. Further, the panels demonstrate sensitivity, reproducibility, and biological fidelity, with sample-type differences reflecting distinct cellular compositions and microenvironments.

## General notes and troubleshooting

**General notes**

1. Barcoding kits and metal-conjugated antibodies used for CyTOF are significantly more expensive than fluorophore equivalents.

2. Limited access to CyTOF systems require specialized infrastructure and a specific set of skills to operate.

3. Traditional manual gating that relies on data visualization using 2D or 3D plots is increasingly difficult with increased parameters for simultaneous assessment. The number of bivariate plots increases significantly, which makes it difficult to manage and is subjective. For this reason, important cell populations of small phenotypic shifts may be easily missed.

4. Effective data analysis requires specialized programs (algorithms) involving dimensionality reduction and unsupervised clustering, which require knowledge regarding computation that is not readily available.

5. Processing and analyzing large CyTOF datasets is computationally intensive and requires significant computing power and specialized software.

6. The Helios is a low-throughput instrument that operates at a maximum acquisition rate of 350 events per second, with a variation of ±50 events per second. Running at higher speeds may introduce clogs and skew results.

7. Unlike flow cytometry, there is no real-time cell sorting on mass cytometers.

8. Instrument drift may occur over time, introducing data variability.

9. The high-dimensional nature of CyTOF may reveal unknown or previously uncharacterized cell subsets or subtle shifts, raising questions about the biological significance of these rare or novel populations. Validating these discoveries often requires the use of orthogonal methods.

**Troubleshooting**

**Problem 1:** Infeasibility of manual bivariate analysis in high-dimensional datasets.

**Possible cause:** Exponential increase in bivariate plots as parameters increase, which makes it difficult to comprehensively identify all relevant cellular populations and phenotype differences.

**Solution:** Employ dimensionality reduction to visualize the data [i.e., t-distributed stochastic neighbor embedding (t-SNE), Uniform Manifold Approximation and Projection (UMAP), principal component analysis (PCA), unsupervised clustering algorithms (FlowSOM, PhenoGraph, Spanning-tree Progression Analysis of Density-normalized Events (SPADE)], and/or open-source tools like R/Bioconductor or Python.

**Problem 2:** Presence of substantial nonspecific binding.

**Possible cause:** Issues with reagent specificity or sample preparation.

**Solution:** Perform antibody titrations to optimize concentrations. Add Fc receptor blocking to prevent immune cells (monocytes, B cells, NK cells, some T cells) from nonspecifically binding to the Fc region of antibodies. Optimize staining buffer and conditions by using fresh PFA, ensuring that 100% methanol is used for phospho-epitope detection. Ensure that there is no aggregation in antibodies by filtering prior to staining.

**Problem 3:** Presence of a high proportion of non-cellular events (i.e., red blood cells from whole blood, debris from PBMCs, and dead or dying cells) recorded.

**Possible cause:** Impartial lysis of red blood cells contributing to non-cellular events that may interfere with staining. Cryopreserved cells release DNA from damaged cells when thawed. Dead or dying cells stick together and are prone to nonspecific antibody staining and aggregation contributing significantly to debris.

**Solution:** Ensure complete lysis of RBCs using the thaw/lyse reagent prior to barcoding. In the context of PBMCs, ensure that density gradient centrifugation is meticulous to minimize contamination of granulocytes or other debris. For PBMCs that are being thawed, use DNase I to reduce aggregates from dead/dying cells that have released DNA. Include cell-ID cisplatin for live/dead gating so these cells can be gated out when analyzing.

**Problem 4:** Insufficient cell numbers to acquire the samples on the Helios.

**Possible cause:** Most cell loss occurs during the cell processing and staining protocol, typically during intracellular staining following methanol treatment.

**Solution:** Barcoding and pooling cells can help reduce cell loss. Additionally, increasing the centrifugation time after methanol permeabilization to 10 min should help with cell yield.

**Problem 5:** Suboptimal phospho-epitope intensity.

**Possible cause:** Incorrect formaldehyde concentration or timing, inadequate permeabilization for intracellular antibody access, incorrect antibody concentrations, use of degraded or expired reagents.

**Solution:** Optimize the whole blood fixation protocol for rapid and complete cross-linking. Utilize ice-cold 100% methanol for permeabilization to ensure proper membrane disruption. Titrate all intracellular phospho-markers using fresh samples before executing a run of the full panel. Check the expiration date and stability of all phospho-antibodies and reagents.

**Problem 6:** Loss of cells following methanol permeabilization.

**Possible cause:** Inadequate centrifugation speed/duration or cell loss following methanol permeabilization.

**Solution:** Increase centrifugation speed/duration after methanol permeabilization (i.e., 1,000–1,200× *g* for 10 min) to ensure complete cell pelleting.

**Problem 7:** Heterogeneous stimulation responses across donors.

**Possible cause:** Inherent biological donor variability or lack of standardization of stimulation.

**Solution:** Standardize critical parameters including stimulation kinetics, incubation temperature, and precise stimulant concentrations. Include known reference stimuli to, e.g., IFNα or LPS, which serves as a vital internal control to confirm the integrity of the assay's stimulation capacity.

**Problem 8:** Challenges with data reproducibility across experimental runs.

**Possible cause:** Batch effects arising from variations during the assay or acquisition.

**Solution:** Implement a robust barcoding strategy to mitigate within-experiment batch effects by consolidating samples for concurrent processing and acquisition. For comprehensive batch normalization, ensure that all samples intended for direct comparison are processed on the same day, using consistent reagent lots for all staining mixtures.

**Problem 9:** Cell aggregation and instrument clogging during mass cytometer acquisition.

**Possible cause:** Insufficient removal of unbound reagents, particulate contamination (such as residual RBC debris), or excessive exposure of cells to viability agents like Cell-ID Cisplatin.

**Solution:** Filter all samples immediately before acquisition. Limit the time in CAS+ by processing one sample at a time for acquisition on the Helios. Ensure that RBCs are lysed prior to barcoding and staining.

## Supplementary information

The following supporting information can be downloaded here:

1. Table S1. NIH Clinical Center’s Blood Bank Donor Race Key.

2. Table S2. Healthy whole blood donor information.

3. Table S3. Expected STAT phosphorylation responses to IFNα, IFNγ, and IL-21.

4. Table S4. Expected MAPK & IκBα phosphorylation responses to LPS.

5. Figure S1. High-dimensional immunophenotyping reveals distribution of the total immune subsets in healthy human donors by CyTOF.

6. Figure S2. Optimization of key antibody concentrations on human PBMCs and whole blood via CyTOF.

7. Figure S3. Time-course of IFNα-induced STAT phosphorylation in Jurkat, Raji, and THP-1 cell lines.

8. Figure S4. Validation of cytokine-induced STAT phosphorylation in human PBMCs.

9. Figure S5. Cytokine-induced STAT phosphorylation in cPBMCs.

10. Figure S6. Cytokine-induced STAT phosphorylation in whole blood.

11. Figure S7. Validation of LPS-induced signaling in human PBMCs and whole blood.
